# Graphle: Interactive exploration of large, dense graphs

**DOI:** 10.1186/1471-2105-10-417

**Published:** 2009-12-14

**Authors:** Curtis Huttenhower, Sajid O Mehmood, Olga G Troyanskaya

**Affiliations:** 1 Department of Computer Science, Princeton University, Princeton, NJ 08540, USA; 2 Lewis-Sigler Institute for Integrative Genomics, Princeton University, Princeton, NJ 08540, USA; 3 Current address: Department of Biostatistics, Harvard School of Public Health, Boston, MA 02115, USA

## Abstract

**Background:**

A wide variety of biological data can be modeled as network structures, including experimental results (e.g. protein-protein interactions), computational predictions (e.g. functional interaction networks), or curated structures (e.g. the Gene Ontology). While several tools exist for visualizing large graphs at a global level or small graphs in detail, previous systems have generally not allowed interactive analysis of dense networks containing thousands of vertices at a level of detail useful for biologists. Investigators often wish to explore specific portions of such networks from a detailed, gene-specific perspective, and balancing this requirement with the networks' large size, complex structure, and rich metadata is a substantial computational challenge.

**Results:**

Graphle is an online interface to large collections of arbitrary undirected, weighted graphs, each possibly containing tens of thousands of vertices (e.g. genes) and hundreds of millions of edges (e.g. interactions). These are stored on a centralized server and accessed efficiently through an interactive Java applet. The Graphle applet allows a user to examine specific portions of a graph, retrieving the relevant neighborhood around a set of query vertices (genes). This neighborhood can then be refined and modified interactively, and the results can be saved either as publication-quality images or as raw data for further analysis. The Graphle web site currently includes several hundred biological networks representing predicted functional relationships from three heterogeneous data integration systems: *S. cerevisiae *data from bioPIXIE, *E. coli *data using MEFIT, and *H. sapiens *data from HEFalMp.

**Conclusions:**

Graphle serves as a search and visualization engine for biological networks, which can be managed locally (simplifying collaborative data sharing) and investigated remotely. The Graphle framework is freely downloadable and easily installed on new servers, allowing any lab to quickly set up a Graphle site from which their own biological network data can be shared online.

## Background

As the breadth, depth, and quantity of biological data has continued to grow, these data have increasingly been represented as graphs or networks for the purposes of analysis and visualization. Historically, biological networks have been used to represent the organization of metabolic pathways [1], protein complexes [2,3], and regulatory networks [4,5], often based on laboratory work carried out before the advent of high-throughput technologies. With the introduction of genome-scale data, datasets from protein-protein interaction networks (PPIs, [6,7]) to microarray correlations [8,9] have all been represented as graphs; computational predictions including regulatory networks [10,11] and functional relationships [12,13] are generally presented as network structures as well. Most commonly, each vertex indicates a gene and each edge a biological relationship, weighted or unweighted (e.g. expression correlation versus PPIs) and undirected or directed (e.g. PPIs versus regulator/target interactions). Not only do graph structures represent a well-understood computational platform for the analysis of these networks on a whole-genome scale [14], they offer a rich visual representation of the varied molecular interactions underpinning systems biology.

The visualization of biological networks has inspired substantial research and tool development, ranging from the detailed organization of small, sparse networks as pathways (e.g. Cytoscape, Osprey, VisANT, and others [15-18]) to visual overviews of entire genomes [19]. Another class of online tools focus on visualization of multiple network alignments [20,21]. Unfortunately, many biological networks of interest fall between these two extremes of size and detail. Genomic data is often large (most organisms of interest have tens of thousands of genes), but not so large that it falls into the class of "huge" network visualization (e.g. maps of the Internet, with some half a billion current hosts); tools for exploring such tremendous networks typically hide the details that are vital for understanding biological networks. Similarly, while many types of biological networks have a small-world-like property [22] and are thus relatively sparse, other graphs are dense or even fully connected (e.g. microarray correlations); standard visualizations of such graphs usually degenerate into uninformative "hairballs" [23]. Moreover, regardless of network size, useful biological graph visualizations must allow for wide variation in scale and detail: most biologists, when presented with a biological network, want to see both the big picture and the specific interactions surrounding their gene(s) of interest. This introduces a need for biological network visualization that appropriately balances scalability, interactivity, and specificity of data presentation.

**Figure 1:**
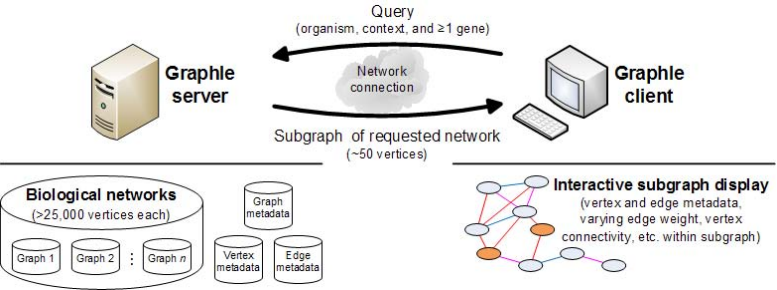
**Overview of the Graphle system architecture**. The Graphle server manages up to hundreds of gigabytes of weighted undirected graphs; while any graph data can be used, Graphle is specifically designed for biological networks in which vertices represent genes and edges represent experimental results (microarray correlations, protein-protein interactions, etc.) or computational predictions (e.g. probabilities of functional interactions). The server also associates metadata with graphs (such as what organism or biological context they are drawn from), vertices (gene identifiers, aliases, known cellular functions, etc.), and edges (e.g. what experiments or data contributed to that edge). The Graphle client communicates user-provided queries to the server consisting of one or more genes of interest, receives an appropriate subgraph, and displays it interactively for the user in a web browser. The user can then change the focused genes or the stringency cutoff for vertex or edge weights and can access the associated metadata to interactively explore tractable portions of the large underlying graphs.

We have created Graphle as a tool to address these issues and to provide biologists with a tool for exploring large biological networks. As shown in Figure 1, Graphle consists of two parts, the main one being a Java-based client that runs in a user's web browser to display interactive, controllable portions of large biological networks (as well as associated data on genes, protein functions, and experimental datasets). This client allows a user to navigate within a biological network either horizontally, by focusing different sets of one or more query genes and viewing their network neighborhood, or vertically, by limiting the view to more or less heavily weighted edges and vertices. For example, if edge weights represent microarray correlations, this allows a user to view only the most correlated pairs of genes or to decrease the cutoff and see additional, weaker relationships. Underlying the Graphle client is a server that can run in a centralized location to manage up to hundreds of biological networks, possibly representing several hundred gigabytes of data. Communication between the server and client is optimized so that only the small, focused portions of the underlying networks surrounding a user's query are communicated to the client, which in turn fine-tunes the visualization of this subgraph. Graphle thus allows a user to flexibly explore any biological network and to interactively scale between very general and very detailed visualizations of specific genes of interest. An implementation of Graphle is available online at http://function.princeton.edu/graphle, showing functional relationship networks predicted for *S. cerevisiae *by the bioPIXIE system [24], for *E. coli *by the MEFIT system [25], and for human beings by the HEFalMp system [26]; a downloadable Java implementation with source code and documentation are also available at this address.

## Implementation

Graphle is implemented in Java using a client/server architecture to modularize the two main components of the system: a graph server that manages a (potentially very large) collection of weighted graphs and associated metadata, and a user interface client that provides an interactive visualization of portions of this data. This partitions the system to allow hundreds of gigabytes of biological network data to be managed on the server while still providing a focused, responsive user experience. The responsibilities of the graph server include accessing large amounts of graph data on disk in a query-driven manner, caching this data to improve performance, executing graph query algorithms based on client input, and providing information on genes (vertices) and underlying data (edges) as needed. The graph client must run in a web browser and provide rapid, interactive access to all data managed by the server in an informative visualization. Fundamentally, just as Google acts as a query-driven server to present an informative subset of the web, the Graphle server acts in a query-driven manner to filter and present the content of biological networks.

### Graph server

The Graphle server is based on a Java port of portions of the Sleipnir C++ library for computational genomics [27] that allow it to efficiently manage multiple large biological networks. Subgraphs are retrieved from these networks using any graph query algorithm. The bioPIXIE [13] and HEFalMp [26] algorithms are currently implemented and can be configured in the server; the former selects high-scoring genes based on their total connection weight to all query genes. The HEFalMp algorithm scores each gene by the ratio of its average connection weight to the query genes over its total average connection weight. Regardless of graph query algorithm, the resulting neighborhood is communicated to the client using a standard socket connection. The graph data organized by the server can include continuous or discrete experimental results (e.g. pairwise correlations from microarray data or protein-protein interaction networks), predicted interaction networks, ontological structures such as the Gene Ontology [28], or any undirected weighted (or unweighted) graphs.

Graph data is stored using the Sleipnir CDat interface, and can thus be interconverted between human-readable text (referred to as the DAT format) and a compact binary (DAB) format. Graphs stored as DABs are automatically indexed and memory mapped; due to memory mapping restrictions on many platforms, an LRU cache is used to maintain a subset of currently mapped graphs. Retiring a graph from this cache, loading a new ~25,000 gene graph, and performing a complete graph query takes at most ~20 s on a modern server, most of which is spent in disk access.

The graph server also maintains metadata describing graphs, vertices, and edges. Each graph is assigned to a particular organism (or other broad category) and to a "context" within that organism, where a context can be a biological process, tissue type, or other specific subcategory. Vertices are described by a unique identifier (e.g. ORF IDs for yeast genes, HGNC [29] symbols for human genes, etc.) and zero or more synonymous aliases; they may also possess zero or more categories of metadata, with each category consisting of an arbitrary dictionary of key/value descriptors (e.g. textual descriptions, Gene Ontology annotations, etc.) Similarly, edges may also be decorated with arbitrary category dictionaries of metadata; this is particularly useful in the case of graphs representing predicted biological networks, as it provides a convenient way to indicate what experimental data was integrated to produce each predicted interaction [13].

### User interface client

The Graphle client is a Java applet designed to interactively visualize configurable subgraphs of biological networks (or other graph data) in a web browser. The client uses the Prefuse library http://prefuse.org for graph layout, supplementing it with an interface for selecting organisms and contexts, displaying vertex/edge metadata, exporting image or text representations of the current graph, and performing graph queries. These queries consist of a user-provided set of genes (or other vertex identifiers) sent to the Graphle server, which performs a configurable graph query algorithm to return the most relevant portion of the selected (potentially very large) complete graph. In addition to controlling which genes make up the current query, the client also provides realtime filters for vertex and edge inclusion (based on the weight of the graph's edges and the confidence with which the server indicates that vertices are included in the graph query results). The combination of these three features allows a user to fluidly and tractably navigate through large, dense, weighted graphs.

## Results

Graphle provides a web-based system for interactively browsing large biological networks. These graphs can represent experimental results (e.g. protein-protein interaction networks, microarray correlations, etc.), computational predictions (e.g. probabilities of functional interaction), or any other undirected, weighted graphs. Each underlying graph can be very large (tens of thousands of vertices, hundreds of millions of edges, gigabytes of data), and the Graphle server can manage hundreds of such graphs along with associated metadata (organism, biological context, gene, and dataset descriptors). The Graphle client executes in a user's web browser and retrieves subgraphs focused on a specific set of query genes. This query and the displayed subgraph can be interactively modified in realtime, allowing a user to conveniently explore targeted subgraphs of interest extracted from the large body of underlying data.

### Graph queries and exploration

**Figure 2:**
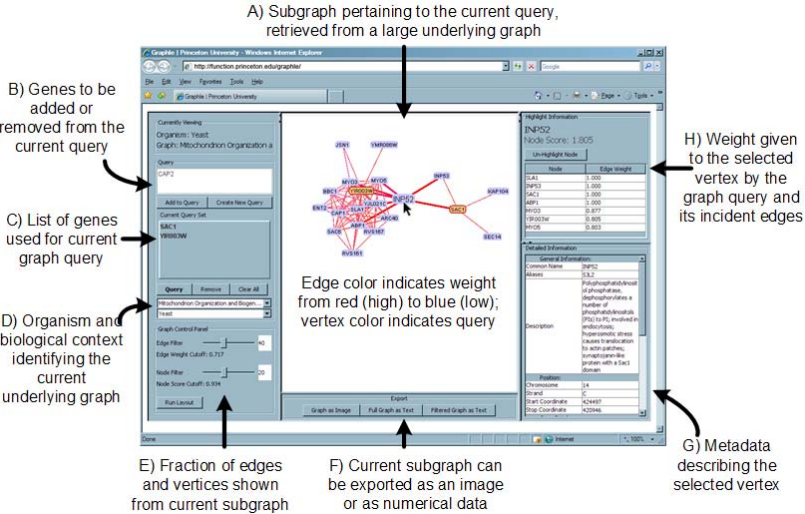
**The Graphle client user interface**. A user can specify one or more genes that are sent as a query to the server. This information allows the server to execute a graph query in the underlying large biological network specified by the requested organism and biological context. A subgraph comprising ~50 vertices total is returned to the client, which then lays out and displays in real time the most informative portion of this subgraph. The visible subgraph can be controlled by modifying the edge and vertex cutoffs. Detailed information on the numerical scores of the selected vertex and its incident edges are shown on the right. The current subgraph can be exported as an image (e.g. for publication) or as raw data (e.g. for further analysis).

A Graphle query consists of two components: a particular underlying graph specified by an organism and biological context (Figure 2D), and one or more gene identifiers specific to that organism (Figure 2B and 2C). For example, a Graphle server may have access to several graphs, each covering a specific context in yeast, human, mouse, or another organism's data; contexts represent variables such as biological processes (the cell cycle, apoptosis, glucose metabolism, etc.), tissue or cell types, or developmental stages. A user of the Graphle client selects an organism and context from the server-provided list and queries on one or more of the organism's genes. These genes are sent to the server, which uses a configurable graph query algorithm [13,26], described above) to select the subgraph of the requested network most relevant to the query genes (Figure 2A). This subgraph is of sufficiently small size (~50 fully connected vertices and the associated edge weights) to be sent to the client in full; the client then provides a configurable visualization of the subgraph for the user.

Edge weights in biological networks often represent the strength of our confidence in an experimental outcome: greater sequence similarity, higher correlation between gene expression values, or larger probabilities of functional interactions, for example. Similarly, using the concept of guilt by association, most graph query algorithms assume that vertices more strongly connected to the query set in the aggregate are in turn more biologically related to those query genes. Correspondingly, the Graphle client allows a user to fine-tune the visualization of a queried subgraph by filtering edges by weight and vertices by score (Figure 2E); filter changes automatically rerun the graph layout algorithm, which is animated to maintain visual context. A biologist can thus easily visualize both strong and diffuse clusters in the data, expand from the most related genes to more distant neighbors, and easily track the relationship(s) of the original query genes to these neighbors.

### Using Graphle: investigating genes and sharing data

A typical use of Graphle is for a biologist to investigate specific genes in a pre-existing biological network. For example, suppose a yeast biologist is interested in the roles of SAC1 (a known regulator of the actin cytoskeleton found in the mitochondrial membrane [30,31]) and the uncharacterized ORF YIR003W in the process of mitochondrion organization and biogenesis. Using the Graphle query shown in Figure 2, an investigator can obtain a visualization of functional interactors (Figure 2A) as predicted by the bioPIXIE system [24]. The number and minimum confidence of the displayed interactors can be controlled interactively (Figure 2E), and the data used to make the predictions (Figure 2G) and their confidences (Figure 2H) are shown directly within Graphle. From this, one might conclude that YIR003W likely participates in cytoskeletal processes through a variety of potential interaction partners (MYO3, MYO5, ABP1, ARC40, etc.)

Conversely, a biologist who generates a large interaction dataset or a bioinformatician with predicted interaction networks can share their data online using Graphle. Particularly for higher eukaryotes with large genomes, the data for a single interaction network can be gigabytes in size; when tens or hundreds of such networks are predicted, transmitting them en masse becomes impractical. A Graphle server can be paired with any web server to share new data for collaborators to query and explore, with few limitations on graph size; the Graphle installation at http://function.princeton.edu/graphle shares approximately 350 GB of biological networks. The process for creating a new Graphle server installation is also detailed on this web site.

### Multiple organisms and biological contexts

The Graphle server organizes its collection of graphs using two biologically motivated levels of abstraction: each graph is assigned to exactly one organism and one biological context (Figure 2D). A graph's organism dictates what unique gene identifiers (and non-unique gene aliases) are used to label its vertices, since the server maintains sets of known genes specific to each organism. A context, practically speaking, can be any unique identifier of a particular graph; in practice, a context is often the experiment that generated the graph data, the computational algorithm that generated a set of predictions, a specific biological system (cell/tissue type, pathway or process, subcellular compartment, etc.), or a combination of these. For example, the Graphle system running at http://function.princeton.edu/graphle offers graphs generated by bioPIXIE [24] in yeast, MEFIT [25] in *E. coli*, or HEFalMp for human data [26], with contexts representing different biological processes on which the two algorithms focused.

### Gene (vertex) and data (edge) information

Graphle maintains arbitrary metadata optionally describing each vertex (gene) and edge in its graphs (Figure 2G). For genes, this metadata is most often useful for conveying standard knowledge associated with genes: synonymous gene identifiers, chromosomal location, known functions cataloged in the Gene Ontology [28] or elsewhere, etc. For edges, this metadata can provide information on the experimental data underlying the graph visualization. This is most important in graphs representing computational data integrations, since each edge might then summarize information from many experimental results - the specifics of which can be provided in the appropriate edge metadata.

### Exporting graph images and data

Graphle provides the opportunity for users to export the current subgraph as an image or as raw textual data (e.g. for further analysis, Figure 2F). Data exported in this manner is provided as a simple edge list linking unique vertex identifiers (i.e. gene names) with the weight of the edge joining them (the semantics of which are dependent on the source of the underlying graph). The currently visible, filtered subgraph can be exported as an image of quality suitable for publication.

## Conclusions

We present Graphle, a system for interactively exploring large, densely connected biological networks. This task has been particularly challenging in the past due to the impracticalities of storing these graphs (which can each be several gigabytes in size) and visualizing them in an informative manner (as they can be fully connected, but with edge weights varying over a potentially wide range). Graphle allows collections of dense, weighted graphs to be stored on a server and accessed through focused queries by a web-based client. The data comprised by such graphs can range from experimental results to computationally predicted interaction networks, and Graphle allows each vertex (i.e. gene) and edge to be annotated with arbitrary descriptive metadata. A web-based client sends sets of query genes from a user to the server and interactively displays the resulting focused subgraphs, which can be manipulated in realtime and exported as data for analysis or as images for publication. The Graphle source code, documentation, and a demonstration client can be found at http://function.princeton.edu/graphle. Graphle thus provides a complete solution for storing, sharing, and exploring biological networks.

## Availability and Requirements

*Project name*: Graphle

*Project home page*: http://function.princeton.edu/graphle

*Operating system(s)*: Platform independent

*Programming language*: Java

*Other requirements*: Java 1.5 or higher

*License*: Creative Commons Attribution 3.0

*Any restrictions to use by non-academics*: No

## Authors' contributions

SOM and CH implemented the software. CH designed the software and drafted the manuscript. OGT coordinated the study. All authors read and approved the final manuscript.
